# Aptamer-Modified Semiconductor Quantum Dots for Biosensing Applications

**DOI:** 10.3390/s17081736

**Published:** 2017-07-28

**Authors:** Lin Wen, Liping Qiu, Yongxiang Wu, Xiaoxiao Hu, Xiaobing Zhang

**Affiliations:** 1 Molecular Science and Biomedicine Laboratory (MBL), State Key Laboratory of Chemo/Bio-Sensing and Chemometrics, College of Chemistry and Chemical Engineering, Hunan University, Aptamer Engineering Center of Hunan Province, Changsha 410082, China; mumuredhouse@hnu.edu.cn (L.W.); qiuliping@hnu.edu.cn (L.Q.); wuyongxiang@hnu.edu.cn (Y.W.); 2 College of Life Sciences, Molecular Science and Biomedicine Laboratory (MBL), State Key Laboratory of Chemo/Bio-Sensing and Chemometrics, Hunan University, Aptamer Engineering Center of Hunan Province, Changsha 410082, China

**Keywords:** semiconductor quantum dots, aptamer, biosensors

## Abstract

Semiconductor quantum dots have attracted extensive interest in the biosensing area because of their properties, such as narrow and symmetric emission with tunable colors, high quantum yield, high stability and controllable morphology. The introduction of various reactive functional groups on the surface of semiconductor quantum dots allows one to conjugate a spectrum of ligands, antibodies, peptides, or nucleic acids for broader and smarter applications. Among these ligands, aptamers exhibit many advantages including small size, high chemical stability, simple synthesis with high batch-to-batch consistency and convenient modification. More importantly, it is easy to introduce nucleic acid amplification strategies and/or nanomaterials to improve the sensitivity of aptamer-based sensing systems. Therefore, the combination of semiconductor quantum dots and aptamers brings more opportunities in bioanalysis. Here we summarize recent advances on aptamer-functionalized semiconductor quantum dots in biosensing applications. Firstly, we discuss the properties and structure of semiconductor quantum dots and aptamers. Then, the applications of biosensors based on aptamer-modified semiconductor quantum dots by different signal transducing mechanisms, including optical, electrochemical and electrogenerated chemiluminescence approaches, is discussed. Finally, our perspectives on the challenges and opportunities in this promising field are provided.

## 1. Introduction

**Figure 1:**
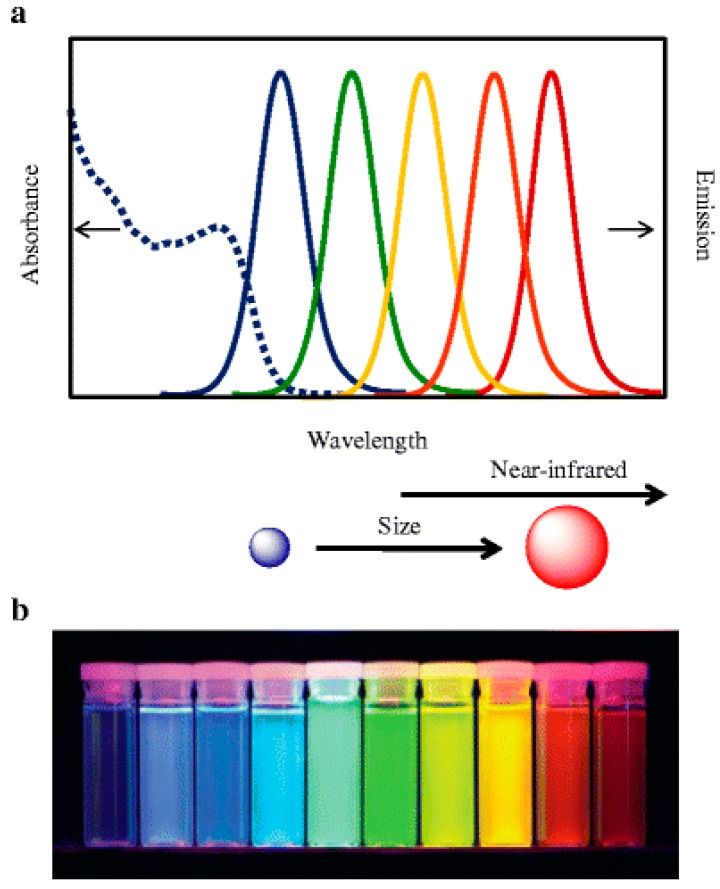
(**a**) Size-tunable emission spectra of QDs; (**b**) 10 distinguishable emission colors of ZnS-capped CdSe QDs by a near-UV lamp. From left to right (blue to red), the emission maxima are located at 443, 473, 481, 500, 518, 543, 565, 587, 610, and 655 nm [6].

Quantum dots (QDs) are colloidal nanocrystalline semiconductors and possess properties such as a quantum confinement effect. The three major kinds of QDs include the homogeneous structures (CdTe, CdSe), core-shell structures (CdTe/CdS), and ternary structures (CdTeSe). QDs with particle sizes of 1–10 nm, close to or less than the exciton of the Bohr radius, with significant quantum effects [1,2], provide a new type of nanomaterial with unique optical and electronic properties. The excellent optical properties include: (1) Broad and consecutive excitation bands, high absorptivity [3]; (2) Narrow and symmetric emission, long fluorescence lifetime; (3) Good photostability [4], with low susceptibilities to photo-bleaching; (4) Tunable emission wavelength (Figure 1) [5,6,7]. These features make them fluorescent probes with unparalleled advantages compared to traditional organic dyes [1,8]. In recent years, quantum dots have been widely applied as fluorescent probes in the biological field.

The electrical properties of quantum dots include: high band-gap, high absorption coefficient and large intrinsic dipole moments [9]. Therefore, in the visible region, they can effectively capture light energy, produce electron-hole pairs, quickly separate electrons and holes, and reduce the electronic loss, so that they can quickly and effectively implement the optical-electrical signal, conversion and electrical signal transmission. These features make them unique and excellent tools in electrochemical and photo-electrochemical sensing. In addition, the conjugation of functional ligands is expected to be essential for QD-based targeted biosensing applications. However, the semiconductor nanomaterials cannot specifically recognize the target individually and need to be combined with a target recognition module.

Aptamers are artificial oligonucleotides (DNA or RNA) and can bind to various entities (e.g., metal ions and etc.) with high affinity and specificity, equal to or superior to those properties of antibodies [10,11]. These aptamers can be identified from combinatorial nucleic acid libraries by in vitro selection methods [12]. Aptamers have several advantages, including easy synthesis, low cost, small size and convenient modifications with various functional groups and nanomaterials. Moreover, proteins tend to be irreversibly denatured in certain conditions, while aptamers are capable of reversible denaturation.

To modify aptamers onto ODs, four major strategies can be used: (1) Self-assembly between DNA and QDs [13,14,15,16]. Mattoussi et al. firstly used the His6-tagged DNA and dihydrolipoic acid (DHLA)–QD self-assembly, producing compact QD–DNA conjugates [13]. Some alternative modification strategies can also be used in compact QD–DNA conjugates, such as self-assembly of thiolated-DNA onto 3-mercaptopropionic acid (MPA)–QDs [17]. (2) Biospecific interactions, e.g., biotin–avidin (or streptavidin) interaction [18,19,20,21,22]. Biotin can be linked to aptamers through its carboxyl group without affecting their activity. Avidin consists of four identical subunits, which theoretically means an avidin molecule can bind to four biotin molecules with strong binding specificity and affinity. The method has the advantages of high specificity, wide workable temperature and pH range, so it is widely used in the construction of biosensor interfaces. (3) Covalent interactions [14,15,23,24,25]. Aptamers can be covalently bound to chemical groups (e.g., hydroxyl groups, etc.) using well-established bioconjugation reactions. (4) Nucleic acid hybridization. The aptamer terminal is extended to a small section of the nucleotide sequence, and the aptamer functionalization can be achieved through hybridization of the extended nucleotide sequence with its complementary sequence modified on the surface of the nanomaterials.

The combinations of QDs and aptamers are expected to provide various detection platforms, including optical, electrochemical and electrochemical luminescence [26], brings more opportunities for bioanalysis (Table 1). The detection of a variety of analytes, such as proteins, small molecules and tumor cells is available. Here, we summarize recent advances of aptamer-functionalized QDs in biosensing applications. We also discuss the properties and structure of QDs, and the application of biosensors based on aptamer-modified QDs in the context of different signal transducing mechanisms, and further our perspectives are provided on the challenges and opportunities in this promising field.

## 2. Aptamer-Modified QDs for Optical Detection

**Figure 2:**
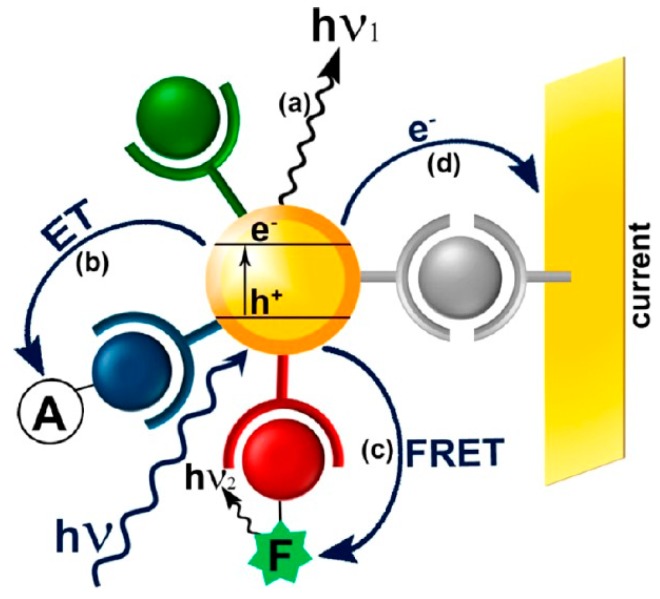
Schematic representation of different QD-based sensing configurations [26]. (**a**) luminescence, (**b**) electron transfer, ET, (**c**) Förster resonance energy transfer, FRET, or (**d**) photocurrent generation.

The excellent optical properties of semiconductor QDs are due to the photoexcitation of the QDs and the transfer of valence-band electrons to the conduction band. Different photophysical fluorescent and chemiluminescence mechanisms (as schematically outlined in Figure 2), combined with specific recognition of aptamers, are used to develop biosensor systems for proteins and small molecules [26].

### 2.1. Fluorescence Detection

QDs have been widely used in aptamer-based biosensors for their fluorescent properties. An on-chip aptamer-based luminescence assay was developed for protein detection by Remcho and co-workers [27]. They used two DNA thrombin aptamers that recognized two different epitopes of thrombin and bound them to different nanoparticles. Aptamer-functionalized magnetic beads were then used to capture the target (thrombin) and another aptamer-functionalized QDs were employed as fluorescent labels for signal generation. In the presence of thrombin, the two aptamers bind to thrombin to form a sandwich, which is then monitored by fluorescence microscopy. The sandwich assay was carried out with disposable microfluidic devices, which were fabricated on double-sided tapes and polymeric materials by a laser cutting approach. This new approach demonstrated the potential of application in fundamental research or protein analysis for diagnosis. Hua et al. used QDs/SiO_2_ nanoparticles synthesized by coating QDs on the surfaces of monodispersed silica nanoparticles (SiO_2_ NPs), which was beneficial to signal amplification. They demonstrated a novel fluorescent and electrochemical assay to selectively collect and detect MCF-7 cells [28].

**Figure 3:**
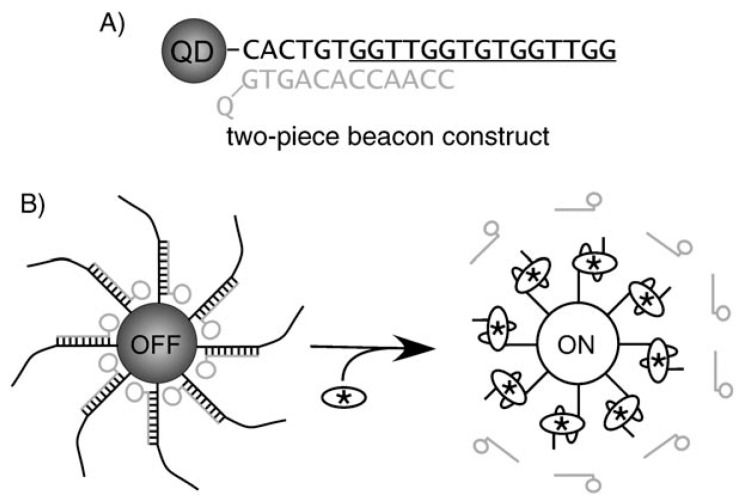
Design of a QD aptamer beacon for the detection of thrombin. (**A**) Anti-thrombin aptamer sequence (black) and quench oligonucleotide (gray). (**B**) Schematic for detection [48].

QDs can be also used in the construction of biosensors by FRET. The first QD-aptamer biosensor on the basis of FRET is reported by Levy et al. [48] (Figure 3). In order to ensure that a substantive conformational change would occur upon analyte binding, they synthesized a typical two-piece beacon which is consisted of a 5-biotinylated anti-thrombin aptamer conjugated to streptavidin-coated Qdot 525 (Qdot Corporation, Hayward, CA, USA) and an 3-quencher-labeled antisense oligonucleotide hybridized to ensure the occurrence of FRET. Meanwhile, to ensure the efficient quenching of quantum dots, multiple aptamer beacon–oligonucleotide quencher pairs were attached to each QD. With thrombin, the aptamer folded into a stabilized quadruplex structure, and resulted in the displacement of the antisense oligonucleotide quencher conjugate, and then caused an increase in Qdot 525 emission. A specific fluorescence response is demonstrated for real-time detection of unlabeled thrombin at 37 °C. But, this design is not optimal, because multiple target molecules (thrombins) are necessary to overcome the QD quenching, as multiple antisense-quencher strands are bound per QD, which means that very low thrombin concentrations cannot be detected easily.

Some QD-aptamer beacon designs were also reported to realize label-free detection of thrombin [29,49], platelet derived growth factor (PDGF) [33], epithelial tumor marker mucin 1 [30], and cocaine [34,50], respectively.

With various signaling and reporting designs, the reported QD-aptamer beacons were either 2-piece or 3-piece constructs that formed stable structures on QDs and functioned upon target-induced strand displacement. Chi et al. developed a “one-piece” QD-aptamer beacon that is competitive and less costly in clinical experiments [51]. In this way they employed a one-stranded anti-thrombin aptamer probe which was covalently conjugated to Qdot565 (excitation at 570 nm; emission at 602 nm) to develop a label-free biosensing system. The BOBO-3 (peak emission at 565 nm), a DNA intercalating dye, which can intercalate into a double helix and induce a FRET-mediated emission while QD is illuminated at 365 nm, was used. After thrombin meets the aptamer, conformational change is induced and then caused BOBO-3 dissociate from QD-aptamer conjugate. Thus, the FRET-mediated BOBO-3 emission was reduced, which could be used to quantify the thrombin concentration. This strategy achieved a limit of detection (LOD) of 1 nM thrombin ad it is of the best detection limits for QD-aptamer based assays (e.g., 1 M LOD for thrombin in Levy et al. [48]; 1 nM LOD for thrombin in Swain [29]; 0.4 nM LOD for PDGF in Kim et al. [35]; 250 nM LOD for mucin 1 in Cheng et al. [36]; 0.5 M LOD for cocaine in Zhang et al. [37]).

Other “single-piece” QD-aptamer beacons using quenching materials as competitive acceptors in FRET have also been reported. Li et al. bound aptamer-conjugated QDs to graphene oxide (GO) sheets and formed a GO/aptamer-QD ensemble to enable the fluorescence of QDs to be quenched by nano−metal surface energy transfer (NSET) from the QDs to the GO sheets [36]. When target molecules, i.e., Pb^2+^ ions, were present, the conformational change of aptamer induced by Pb^2+^ led QDs to detach from the GO sheet. As a result, the fluorescence of the QDs was recovered. This sensor exhibits a detection limit of 90 pM and high selectivity toward Pb^2+^ in the presence of various metal ions. Wang et al. used near-infrared quantum-dots (NIR-QDs) as the energy donor and oxidized carbon nanoparticles (OCNPs) as the energy acceptor to detect insulin in vivo [31]. Duan et al. realized simultaneous detection of pathogenic bacteria with an aptamer-based biosensor with dual FRET from QDs to carbon nanoparticles (CNPs) [37]. Sabet et al. used Au nanoparticles (AuNPs) and aptamer-conjugated QDs to detect aflatoxin B1 (AFB1) in rice and peanut [35].

A number of innovative optical detection technologies had also been developed. Willner’s group firstly utilized the self-assembly of the “split” aptamer in the presence of target molecules to develop a series of optical bioanalytical assay [32,34]. Taking cocaine detection as an example [34], one of the subunits of anti-cocaine aptamer was linked to CdSe/ZnS QDs, and the other subunit was incorporated with dye that had the ability of quenching fluorescence of QDs by FRET. In the presence of cocaine, the complex was constructed and thus facilitated the detection of cocaine with a detection limit of 1 × 10^−6^ M.

**Figure 4:**
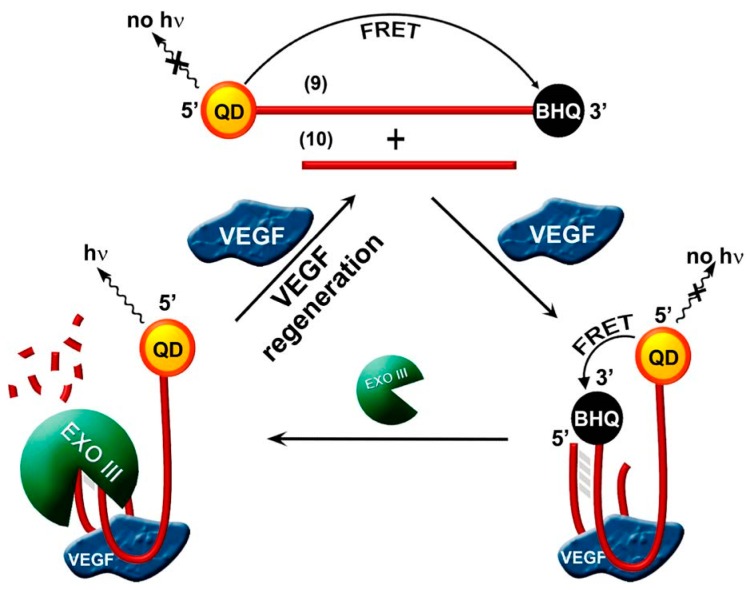
Fluorescence sensing of VEGF is amplified with two aptamer subunits 9 and 10, and Exo III-catalyzed VEGF regeneration [26].

As shown in Figure 4, Willner’s group combined the Exonuclease III (Exo III) recycling amplification assay with self-assembly for vascular endothelial growth factor (VEGF) detection [26,32]. They also developed an amplified optical aptamer sensing system based on the exonuclease III (Exo III) recycling of VEGF. Here the 5′ end and 3′ end of the aptamer is modified with a QD and a black hole quencher, respectively. The VEGF induces self-assembly of the aptamer subunits and caused the digestion of the quencher units and the recycling of the analyte, leading to restoration of the QD luminescence (detection limit 5 pM). In addition, they demonstrated this system could be used to analyze VEGF in human blood. Electron transfer (ET)-based fluorescence quenching of the CdSe/ZnS QDs as a process to develop aptamer-sensors for cocaine and thrombin detection has also been reported by Willner and co-workers [37]. For example, for thrombin detection, the thiolated anti-thrombin aptamer is modified on the glutathione-functionalized CdSe/ZnS QDs by covalent coupling, and then formed a duplex structure with complementary strand, which led to the intercalation of doxorubicin (DB) into the duplex structure and accompanied the quenching of the fluorescence of the QDs. In the presence of thrombin, the structure was unfolded due to the thrombin/aptamer complex was more stable. DB was then removed from the QD nucleic acid and fluorescence was restored. Upon the detection of the protein, this aptamer-sensor led to the turn-on of the fluorescence of the QDs.

### 2.2. Chemiluminescence Detection

#### 2.2.1. Chemiluminescence Resonance Energy Transfer

**Figure 5:**
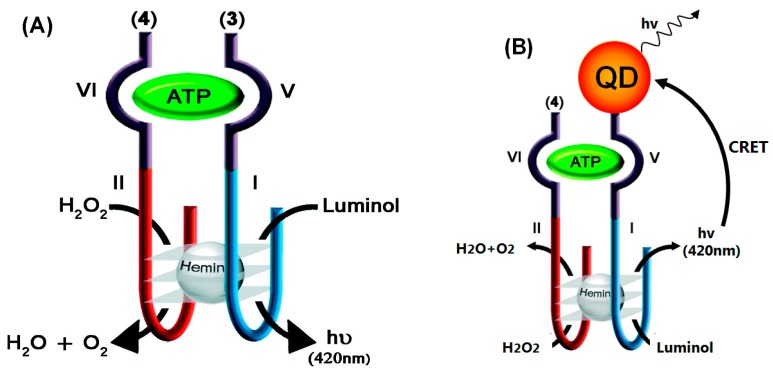
(**A**) Chemiluminescent analysis of ATP. Two subunits are the conjugated anti-ATP and HRP-DNAzyme subunits. (**B**) Analysis of ATP through the CRET from luminol to the QDs [53].

QDs are also used as energy acceptors of energy which is generated by chemiluminescence [52,53]. Research has shown that the hemin/G-quadruplex horseradish peroxidase (HRP)-mimicking DNAzyme can catalyze the oxidation of luminol by H_2_O_2_ to yield chemiluminescence [54]. Willner’s group incorporated hemin and ATP into nucleic acid subunits, including fragments of the HRP-mimicking DNAzyme (I, II) and anti-ATP aptamer domains (IV, V), which self-assembled active DNAzyme and catalyzed the reproduction of chemiluminescence, as shown in Figure 5A. The catalytic processes realized the detection of ATP with detection limits 10 Μm [53]. This method was also can used in detection of thrombin. The DNAzyme stimulated chemiluminescence resonance energy transfer (CRET) to CdSe/ZnS QDs was used to build sensing platforms for detecting ATP or thrombin. Take detection of ATP as an example, the detail was shown in Figure 5B.

One of the nucleic acid subunits reacted with CdSe/ZnS QDs, and thus the self-assembly of the ATP-aptamer subunits/hemin-G-quadruplex DNAzyme caused the CRET signal when ATP and hemin appear. The CRET signals are intensified with the concentration of ATP, due to the higher chemiluminescence signals. In the CRET process, this sensor had an advantage of low chemiluminescence signal background. Finally, the detection limit of ATP was corresponding to 100 nM, which was much lower than with single chemiluminescent detection. At the meantime, this method provided a possibility to design various aptasensor assays with different sized QDs.

#### 2.2.2. Electrogenerated Chemiluminescence

Electrogenerated chemiluminescence (ECL) method includes the generation of species at electrode surface undergoing electron-transfer reactions to form excited states, where light is produced when it decays to the ground state. It is the combination of chemiluminescence (CL) and electrochemistry [55], whose main advantage is that the background interference caused by the excitation light scattering is effectively avoided. This technology integrated the advantages of increased sensitivity and electrochemical potential control for luminescence analysis, and it has become one of the areas of interest for analytical chemists.

Bard and et al. firstly found that QDs could electrogenerated light emission under the potential pulsing or cycling, known as ECL [56,57]. So far, an alternative QDs ECL approach, based on coreactant, has been widely used. When reacting with the co-reactants, efficient and stable ECL in aqueous solution can be obtained by applying a cathodic or anodic potential to the QDs. Therefore, a number of biosensing systems using QDs as ECL labels were developed.

For example, Zhu’s group developed a series of aptasensors with the QDs ECL as the signal transduction for the detection of lysozyme, ATP and thrombin [58,59,60]. Aptasensors detecting lysozyme and ATP were built with the specific affinity between aptamer and target, on the basis of Watson–Crick base pairing. Taking lysozyme detection for example [60], thiolated anti-lysozyme aptamer were modified onto the pretreated Au electrode via an Au-S bond, and then incubated with lysozyme solution to form the aptamer-lysozyme bioaffinity complexes. The free aptamer were hybridized with biotin modified complementary DNA (biotin-cDNA) oligonucleotides. The avidin-modified QDs were bound to the biotin-cDNA. The ECL signal of the biosensor reflected the amount of QDs bonded to the cDNA oligonucleotides, indirectly inversely proportional to the bonded target analyte.

This sensing system can also detect other target molecules by changing the recognition unit. In addition, with incorporation of signal amplification strategies, the detection sensitivity can be further improved. Xie et al. used AuNPs/graphene-modified electrode to build an ECL sensing interface for thrombin detection, and ultrahigh sensitivity was achieved with a detection limit of 10 fM [61]. Jie et al. used a novel dendrimer/CdSe-ZnS-quantum dot nanocluster (NC) as an probe, which amplified the QD’s ECL signal compared with the single QDs linked to aptamer [62]. Meanwhile, a DNA device cycle-amplifying method was developed with greatly improved sensitivity, providing a sensitive and selective detection platform for cancer cells.

Hai et al. constructed a “turn-on” ECL sensor for Pb^2+^ detection using QDs with combination of a G-quadruplex aptamer [41]. Moreover, Liu et al. designed an “off-on” ECL aptasensing method to sensitive detecting ATP with G-quadruplex/hemin wrapped AuNPs to quenching ECL emission of quantum dots, as G-quadruplex had strong catalytic ability to the decrease of dissolved oxygen [40]. Shan et al. reported another amplified ECL quenching mechanism [39]. They used the remarkably efficient energy-transfer between CdS:Mn nanocrystals (NCs) film and CdTe QDs-doped silica nanoparticles (CdTe/SiO_2_ NPs) for the detection of thrombin.

In addition, sandwich structure (aptamer1-target-aptamer2) is constructed for detection. Huang et al. applied QDs ECL in aptasensor field by this sandwich method [60]. Two anti-thrombin aptamers can combine with thrombin to form a stable structure. With thrombin, the sandwich structure (aptamer I-thrombin-aptamer II) could be formed. ECL signal was caused by avidin–QDs which was bound tightly to the aptamer II. In this way, thrombin could be measured with range from 0 to 20 g mL^−1^ and detection limit of 2.72 nM. This biosensor exhibited the potential cycling stability and good selectivity responses to the target, which expands the application of QDs ECL.

Zhang et al. built a new ECL platform for leukemia cell detection by constructing a similar sandwich structure. Unlike the former aptamer I-thrombin-aptamer II structure, they used two identification elements, which were aptamer and concanavalin (con A). A conjugated ZnO@CQDs was used for specific recognition of the cell surface carbohydrates [42]. In the presence of tumor cells, aptamer-cell-con A structure was formed. The use of carbon quantum dots (CQDs)-coated ZnO nanospheres (ZnO@CQDs) enhanced and amplified the ECL intensity and signal. The proposed device has the advantages of high specificity, sensitivity and good stability, and will be a promising tool for sensitive detection of leukemia cells.

Yang et al. covered the tubes of ACNTs with chitosan (CTS)-CdS QDs complex films by electrodeposition reactions between CTS-CdS QDs and an ACNTs electrode [63]. Through this method, they fabricated a CdS QDs/ACNTs electrode with good biocompatibility and high ECL intensity. After anti-thrombin aptamer was bound to the film, this electrode detected thrombin with advantages of high efficiency, sensitivity, specificity and stability. Since thrombin concentration was positively correlated with the level of the ECL intensity, the specific reaction between aptamer and thrombin could reduce ECL intensity. Wang et al. designed enhanced ECL of CdS thin films by ssDNA-AuNP conjugates, with the free aptamer for the detection of thrombin [64]. The system showed 5-fold enhancement of ECL intensity compared to that with no Au NPs. The detectable concentration of thrombin ranged from 100 aM to 100 fM. Li et al. also used water soluble thioglycolic acid (TGA)-modified CdSe QDs, and thus it could bind more aptamer by the carboxyl and the amino groups to improve the ECL signal [65].

## 3. Aptamer-Modified QDs for Electrochemical Detection

### 3.1. Electrochemical Detection without Photo-Excitation

With their great electrochemical properties, QDs act as electroactive species for signal amplification in biosensing. The surface-modified aptamer, with excellent electrical conductivity, is used as a sensitive layer of the electrochemical sensor. For example, Li et al. developed a novel aptamer biosensor for detecting thrombin, and they immobilized aptamer-modified water soluble CdSe QDs on the surface of a glassy carbon electrode (GCE) [65]. With the larger surface area and ion centers of CdSe QDs, the electrochemical signal could be improved. As demonstrated, this constructed biosensor performed better than that without CdSe QDs immobilization.

**Figure 6:**
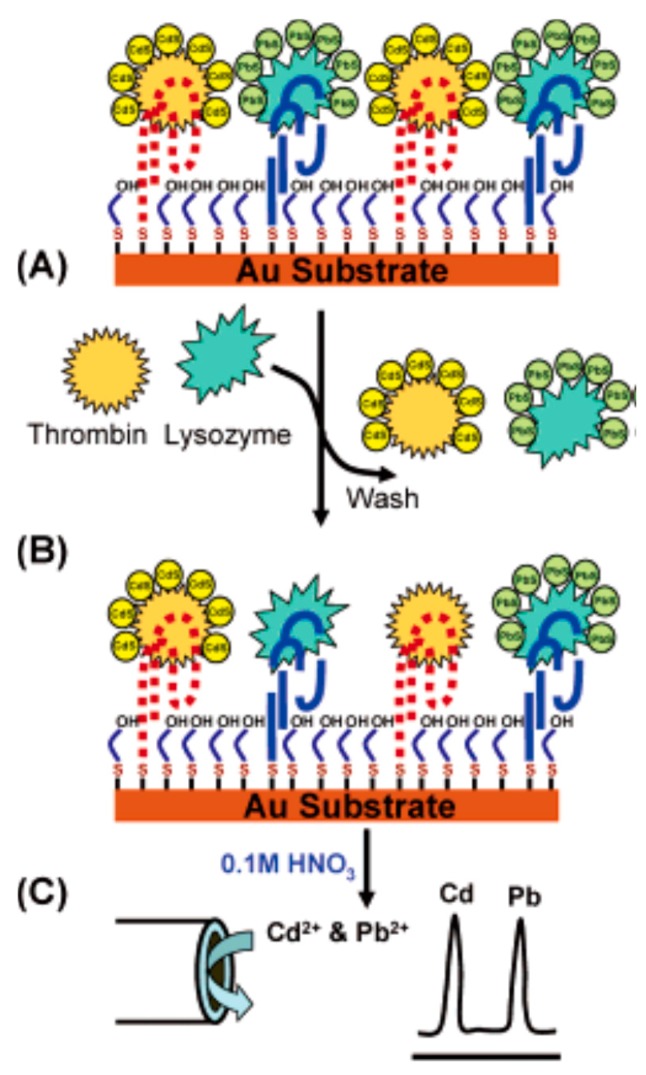
Operation of the aptamer/QD-based dual-analyte biosensor, through displacement of the tagged proteins with the target analytes. (**A**) Monolayer of thiolated aptamers is mixed with the bound protein-QD conjugates; (**B**) The tagged proteins is displaced by the sample; (**C**) The remaining captured nanocrystals are dissolved and apply their electrochemical-stripping detection [43].

After combining aptamers with QDs of high amplification and coding features, Hansen et al. first designed electrochemical biosensors for multi-protein targets with high selectivity and sensitivity [43], as shown in Figure 6. The researchers fixed several thiolated aptamers on a gold surface, which made it possible to capture QD-tagged thrombin or lysozyme. The added protein sample displaced immobilized protein-QD conjugates. Then, they monitored the displacement by the electrochemical stripping detection of the residual nanocrystals. This method provided a detection limit of 0.5 pM. The electrochemical sensing protocol based on interaction of aptamers and protein could detect ultrarace levels of biomarkers and facilitate an early detection of diseases.

Liu et al. built a versatile sandwich strategy for sensitive and selective detection of cancer cells by employing DNA concatamer and QDs as signal amplifier [42]. Firstly, they used MWCNTs@PDA@AuNPs complex as electrode materials to load a lot of concanavalin A. The immobilized Con A was applied to capture cancer cells in high stability. Meanwhile, the trapped cancer cells (CCRF-CEM cells) were detected with aptamer-DNA concatamer-QDs probes by fluorescence and electrochemical methods. This sandwich cytosensor display high sensitivity with the detection limit of 50 cells mL^−1^. Furthermore, it helps separate cancer cells from normal cells, and has promising applications in cancer diagnosis and treatment.

### 3.2. Photoelectrochemical (PEC) Detection

Since combining optical methods and electrochemical sensors can couple photo-irradiation with electrochemical detection, photoelectrochemical (PEC) sensors is a special electrochemical sensing method. To separate the excitation and detection signal, the PEC method showed high signal-to-noise ratios. In PEC detection, signal was based on analytes induced photocurrent change of the photoactive species modified electrode. Thus, the photoactive materials were very important for the performance of the PEC sensors.

Among the photoactive materials, semiconductor QDs, such as CdS, CdSe, PbS and CdTe, which have narrow band gap deposition and can respond to visible light, have received considerable attention in PEC sensing. Upon illumination with photons having energy equaling to or larger than the band gap of the semiconductor, electron–hole pairs are generated, which result in photocurrent. According to the direction of electron transfer on the conduction band, the photocurrent can be divided into anodic photocurrent and cathode photocurrent. When the conduction band electrons are transferred to the electrode material, and the electron donor in the solution transfers the electrons to the valence band holes, an anodic photocurrent is generated. Ascorbic acid (AA) and triethanol-amine (TEOA) are usually exploited as a nontoxic and efficient electron donor. In contrast, when the conduction band electrons are transferred into the solution and combine with the electron acceptor, and the electrons on the surface of the electrode are transferred to the valence band, a cathode photocurrent is generated.

**Figure 7:**
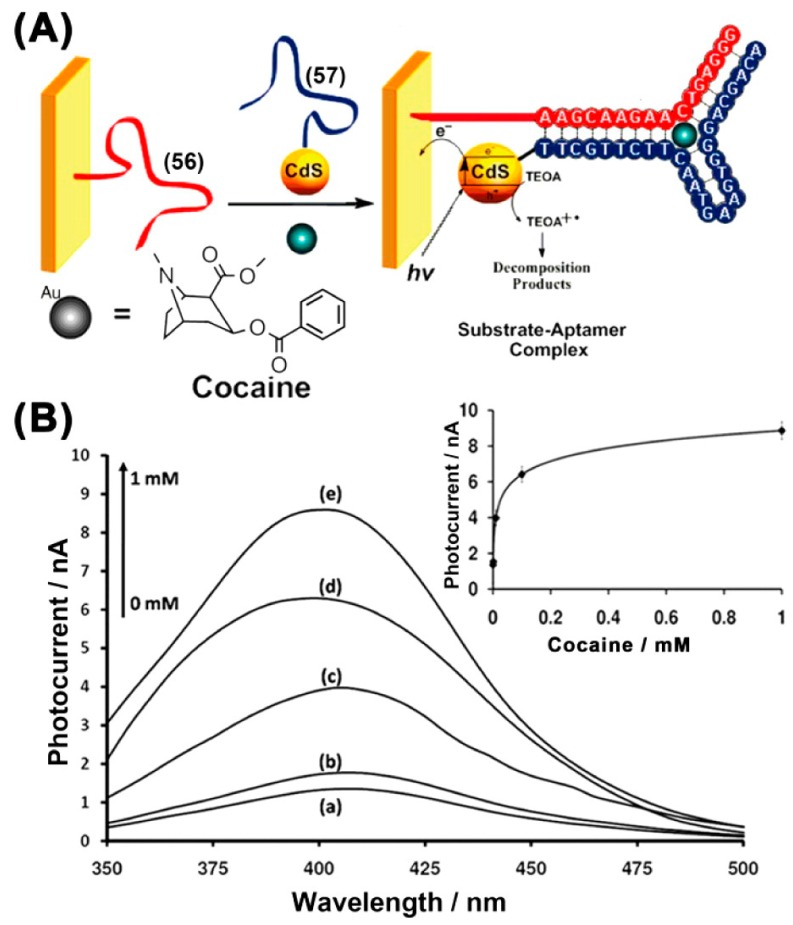
(**A**) Photoelectrochemical cocaine sensor with the self-organization of cocaine/aptamer subunits on an Au electrode. (**B**) Photocurrent action spectra with the analysis of different concentrations of cocaine [46].

Willner et al. developed a PEC detection strategy for cocaine with supramolecular aptamer complexes and semiconductor quantum dots [46]. As shown in Figure 7, they employed CdS QDs as photoactive materials for characterizing the formation of the supramolecular complex between the splitting anti-cocaine aptamer subunits and cocaine. When an electron donor (triethanolamine) appeared, the cocaine complex, CdS-QDs-labeled aptamer subunits, generated photocurrents after being stimulated by light, which enables the PEC detection of cocaine. Due to the excellent photosensitivity of CdS QDs, this method realized the analysis of cocaine with a detection limit to 1 × 10^−6^ M.

Nanocomposites, with the combined advantages of all their materials, can be also applied for PEC biosensing. Tian et al. combined the unique PEC properties of TiO_2_ nanotube (TiO_2_ NTs) arrays with QDs, and built a novel sensitive PEC aptasensor for detecting gene MUC1 [45]. Firstly, the TiO_2_ NTs were fabricated on a titanium foil. Then the gold nanoparticles (AuNPs) were electrodeposited in TiO_2_ NTs tubes, and improved the electrical conductivity of TiO_2_ NTs and bind MUC1 aptamers by the Au–S bond. Subsequently, photosensitizer CdTe QDs were also attached to the TiO_2_ NTs by bingding CdTe QD-labeled c-DNAs to MUC1 aptamers. The MUC1 combined with aptamer competitively. The concentration of MUC1 could be measured by the proposed aptasensor with good reproducibility, low detection limit and stability. Liu et al. synthesized ZnO nanospheres/graphene composite as photoactive materials to improve PEC performance, for the high photoelectric activity of ZnO hollow nanospheres and superior charge transportation and separation of grapheme [47]. Combining with S6 aptamer, it can photoelectrochemically detect SK-BR-3 cells.

## 4. Conclusions and Perspectives

In this review, the applications of aptamer-modified semiconductor quantum dots as hybrid systems for biosensing have been extensively discussed. Different from conventional organic fluorophores or fluorescent proteins, QDs are synthesized by wet chemical synthesis methods. QDs possess many advantages, including broad absorption bands, tunable emission wavelengths, narrow emission bandwidths, and slow photobleaching. After the surface of QDs is modified by one of many flexible functionalization approaches, they can be transformed into a versatile nanoplatform for aptamer conjugation. The prepared aptamer-modified semiconductor QDs bioconjugates can be used for biomedical applications.

The application of hybrid nanostructures is based on the analysis and the use of optical electrochemistry, based in turn on the extensive photophysical mechanisms of QDs, including fluorescence, chemiluminescence and functional recognition of aptamers. Since aptamers can be selected against numerous targets, the aptamer/semiconductor QDs sensors appear to be a promising new sensing platform for broad environmental, biotechnological, and clinical applications.

One important topic in the application of QDs for sensing processes relates to the amplification of the detection platforms. The use of exonuclease III (Exo III), as a biocatalyst for the autonomous regeneration of the target DNA or the aptamer−substrate was discussed. They can be successfully applied for sensing a wide range of molecules, allowing low levels of detection and reduced interference of other compounds in complex samples. However, in view of the size-controlled luminescence properties of QDs, we can expand the detection for different target analytes at the same time. The vast majority of QDs can be only found in bulk solution, which hinders the routine analysis applications so far. Therefore, the future challenge is the innovation of QDs synthesis and conjugation methods, to prepare aptamer/QD-based hybrids with increased stability, sensitivity and binding specificity.

The rapid progress in the application of other functional materials for sensing (e.g., graphene, etc.) and the elucidation of their new optical properties, suggests that by coupling the aptamer/QD-based hybrids with such graphitic carbon nanostructures, novel sensing and detection platforms could be designed. How to incorporate the aptamer/QD-based hybrids into cells and carry out the in vivo sensing of analytes may be a challenge in the future.

## Figures and Tables

**Table 1:** A brief overview of QD-aptamer biosensors.

Biosensor		Representative Targets	Limit of Detection
Optical detection	Fluorescence	Thrombin	0.45 nM [27]
		MCF-7 cells	201 cells mL^−^^1^ [28]
	FRET	Thrombin	1 nM [29]
		Mucin 1	250 nM [30]
		Insulin	0.6 pM [31]
		VEGF	5 pM [32]
		PDGF	0.4 nM [33]
		Cocaine	1 μM [34]
		Aflatoxin B1	3.4 nM [35]
		Pb^2+^	90 pM [36]
	ET	Thrombin	40 μM [37]
		Cocaine	1 μM [37]
	CRET	ATP	100 nM [38]
	ECL	Thrombin	1 aM [39]
		ATP	7.6 nM [40]
		Pb^2+^	10.8 pM [41]
		Leukemia cells	46 cells mL^−1^ [42]
Electrochemical detection	Electrochemical without detection by photoexcitation	Thrombin	0.5 pM [43]
		CCRF-CEM cells	50 cells mL^−1^ [44]
	Photoelectrochemical (PEC) detection	Mucin 1	0.52 nM [45]
		Cocaine	1 μM [46]
		SK-BR-3 cells	58 cells mL^−1^ [47]
